# Physical and Numerical Modeling of the Impeller Construction Impact on the Aluminum Degassing Process

**DOI:** 10.3390/ma15155273

**Published:** 2022-07-30

**Authors:** Kamil Kuglin, Michał Szucki, Jacek Pieprzyca, Simon Genthe, Tomasz Merder, Dorota Kalisz

**Affiliations:** 1 Faculty of Foundry Engineering, AGH University of Science and Technology, Al. A. Mickiewicza 30, 30-059 Krakow, Poland; kamil.kuglin@gmail.com; 2 Foundry Institute, Technische Universität Bergakademie Freiberg, Bernhard-von-Cotta-Str. 4, 09599 Freiberg, Germany; michal.szucki@gi.tu-freiberg.de (M.S.); simon.genthe@gi.tu-freiberg.de (S.G.); 3 Faculty of Materials Engineering, Silesian University of Technology, Krasinskiego 8, 40-019 Katowice, Poland; jacek.pieprzyca@polsl.pl (J.P.); tomasz.merder@polsl.pl (T.M.)

**Keywords:** aluminum, impeller construction, degassing process, numerical modeling, physical modeling

## Abstract

This paper presents the results of tests on the suitability of designed heads (impellers) for aluminum refining. The research was carried out on a physical model of the URO-200, followed by numerical simulations in the FLOW 3D program. Four design variants of impellers were used in the study. The degree of dispersion of the gas phase in the model liquid was used as a criterion for evaluating the performance of each solution using different process parameters, i.e., gas flow rate and impeller speed. Afterward, numerical simulations in Flow 3D software were conducted for the best solution. These simulations confirmed the results obtained with the water model and verified them.

## 1. Introduction

Constantly increasing requirements concerning metallurgical purity in terms of hydrogen content and nonmetallic inclusions make casting manufacturers use effective refining techniques. The answer to this demand is the implementation of the aluminum refining technique making use of a rotor with an original design guaranteeing efficient refining [1,2,3,4]. The main task of the impeller (rotor) is to reduce the contamination of liquid metal (primary and recycled aluminum) with hydrogen and nonmetallic inclusions. An inert gas, mainly argon or a mixture of gases, is introduced through the rotor into the liquid metal to bring both hydrogen and nonmetallic inclusions to the metal surface through the flotation process. Appropriately and uniformly distributed gas bubbles in the liquid metal guarantee achieving the assumed level of contaminant removal economically. A very important factor in deciding about the obtained degassing effect is the optimal rotor design [5,6,7,8]. Thanks to the appropriate geometry of the rotor, gas bubbles introduced into the liquid metal are split into smaller ones, and the spinning movement of the rotor distributes them throughout the volume of the liquid metal bath. In this solution impurities in the liquid metal are removed both in the volume and from the upper surface of the metal. With a well-designed impeller, the costs of refining aluminum and its alloys can be lowered thanks to the reduced inert gas and energy consumption (optimal selection of rotor rotational speed). Shorter processing time and a high degree of dehydrogenation decrease the formation of dross on the metal surface (waste). A bigger produced dross leads to bigger process losses. Consequently, this means that the choice of rotor geometry has an indirect impact on the degree to which the generated waste is reduced [9,10].

Another equally important factor is the selection of process parameters such as gas flow rate and rotor speed [11,12]. A well-designed gas injection system for liquid metal meets two key requirements; it causes rapid mixing of the liquid metal to maintain a uniform temperature throughout the volume and during the entire process, to produce a chemically homogeneous metal composition. This solution ensures effective degassing of the metal bath. Therefore, the shape of the rotor, the arrangement of the nozzles, and their number are significant design parameters that guarantee the optimum course of the refining process. It is equally important to complete the mixing of the metal bath in a relatively short time, as this considerably shortens the refining process and, consequently, reduces the process costs. Another important criterion conditioning the implementation of the developed rotor is the generation of fine diffused gas bubbles which are distributed throughout the metal volume, and whose residence time will be sufficient for the bubbles to collide and adsorb the contaminants. The process of bubble formation by the spinning rotors differs from that in the nozzles or porous molders. In the case of a spinning rotor, the shear force generated by the rotor motion splits the bubbles into smaller ones. Here, the rotational speed, mixing force, surface tension, and fluid density have a key effect on the bubble size. The velocity of the bubbles, which depends mainly on their size and shape, determines their residence time in the reactor and is, therefore, very important for the refining process, especially since gas bubbles in liquid aluminum may remain steady only below a certain size [13,14,15]. 

The impeller designs presented in the article were developed to improve the efficiency of the process and reduce its costs. The impellers used so far have a complicated structure and are very pricey. The success of the conducted research will allow small companies to become independent of external supplies through the possibility of making simple and effective impellers on their own. The developed structures were tested on the water model. The results of this study can be considered as pilot.

## 2. Materials and Methods

Rotors were realized with the SolidWorks computer design technique and a 3D printer. The developed designs were tested on a water model. Afterward, the solution with the most advantageous refining parameters was selected and subjected to calculations with the Flow3D package. As a result, an impeller was designed for aluminum refining. Its principal lies in an even distribution of gas bubbles in the entire volume of liquid metal, with the largest possible participation of the bubble surface, without disturbing the metal surface. This procedure guarantees the removal of gaseous, as well as metallic and nonmetallic, impurities.

### 2.1. Rotor Designs

**Figure 1:**
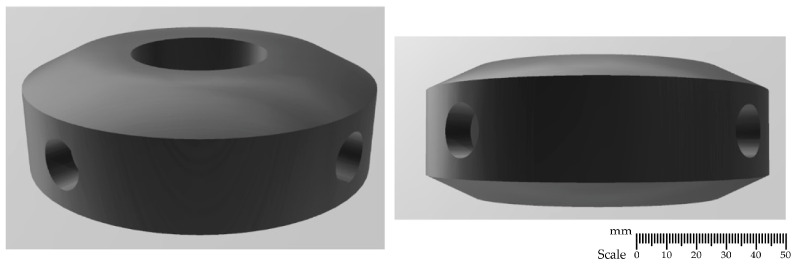
A 3D model—impeller with four holes—variant B4.

**Figure 2:**
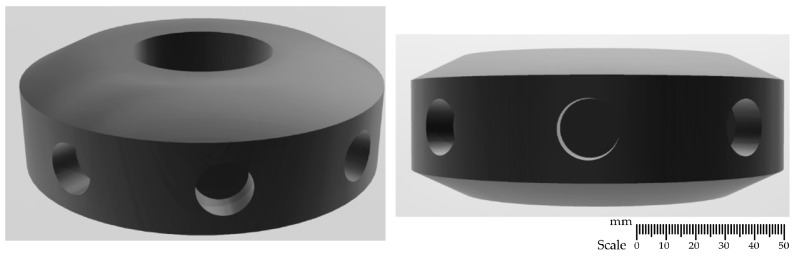
A 3D model—impeller with eight holes—variant B8.

**Figure 3:**
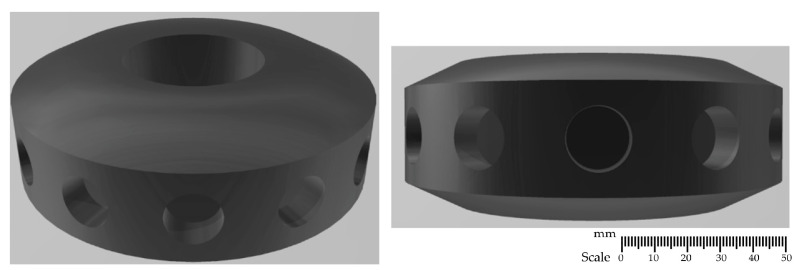
A 3D model—impeller with twelve holes—variant B12.

**Figure 4:**
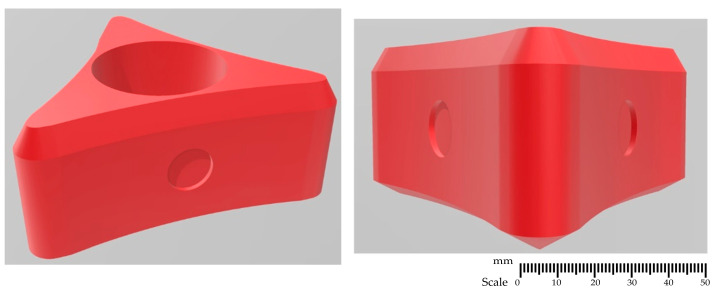
A 3D model—‘red triangle’ impeller with three holes—variant RT3.

The developed impeller constructions, shown in Figure 1, Figure 2, Figure 3 and Figure 4, were printed on a 3D printer using the PLA (polylactide) material. The impeller design models differ in their shape and the number of holes through which the inert gas flows. Figure 1, Figure 2 and Figure 3 show the same impeller model but with a different number of gas outlets. The arrangement of four, eight, and 12 outlet holes was adopted in the developed design. A triangle-shaped structure equipped with three gas outlet holes is presented in Figure 4.

### 2.2. Physical Models

**Figure 5:**
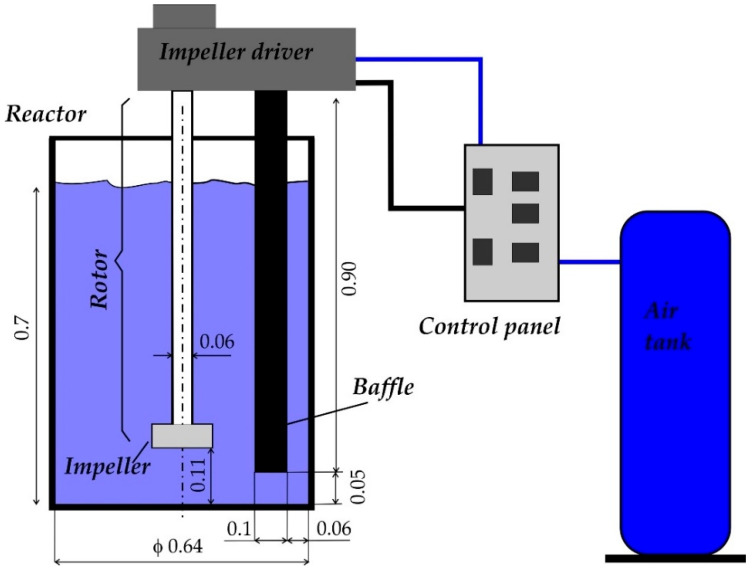
A schematic of the water model of reactor URO 200.

Investigations were carried out on a water model of the URO 200 reactor of the barbotage refining process (see Figure 5). 

The URO 200 reactor can be classified as a cyclic reactor. The main element of the device is a rotor, which ends the impeller. The whole system is attached to a shaft via which the refining gas is supplied. Then, the shaft with the rotor is immersed in the liquid metal in the melting pot or the furnace chamber. In URO 200 reactors, the refining process lasts 600 s (10 min), the gas flow rate that can be obtained ranges from 5 to 20 dm^3^·min^−1^, and the speed at which the rotor can move is 0 to 400 rpm. The permissible quantity of liquid metal for barbotage refining is 300 kg or 700 kg [8,16,17]. The URO 200 has several design solutions which improve operation and can be adapted to the existing equipment in the foundry. These solutions include the following [8,16]:

- URO-200XR—used for small crucible furnaces, the capacity of which does not exceed 250 kg, with no control system and no control of the refining process.
- URO-200SA—used to service several crucible furnaces of capacity from 250 kg to 700 kg, fully automated and equipped with a mechanical rotor lift.
- URO-200KA—used for refining processes in crucible furnaces and allows refining in a ladle. The process is fully automated, with a hydraulic rotor lift.
- URO-200KX—a combination of the XR and KA models, designed for the ladle refining process. Additionally, refining in heated crucibles is possible. The unit is equipped with a manual hydraulic rotor lift.
- URO-200PA—designed to cooperate with induction or crucible furnaces or intermediate chambers, the capacity of which does not exceed one ton. This unit is an integral part of the furnace. The rotor lift is equipped with a screw drive.

Studies making use of a physical model can be associated with the observation of the flow and circulation of gas bubbles. They require meeting several criteria regarding the similarity of the process and the object characteristics. The similarity conditions mainly include geometric, mechanical, chemical, thermal, and kinetic parameters. During simulation of aluminum refining with inert gas, it is necessary to maintain the geometric similarity between the model and the real object, as well as the similarity related to the flow of liquid metal and gas (hydrodynamic similarity). These quantities are characterized by the Reynolds, Weber, and Froude numbers. The Froude number is the most important parameter characterizing the process, its magnitude is the same for the physical model and the real object. Water was used as the medium in the physical modeling. The factors influencing the choice of water are its availability, relatively low cost, and kinematic viscosity at room temperature, which is very close to that of liquid aluminum. 

The physical model studies focused on the flow of inert gas in the form of gas bubbles with varying degrees of dispersion, particularly with respect to some flow patterns such as flow in columns and geysers, as well as disturbance of the metal surface. The most important refining parameters are gas flow rate and rotor speed. The barbotage refining studies for the developed impeller (variants B4, B8, B12, and RT3) designs were conducted for the following process parameters:

- Rotor speed: 200, 300, 400, and 500 rpm,
- Ideal gas flow: 10, 20, and 30 dm^3^·min^−1^,
- Temperature: 293 K (20 °C).

These studies were aimed at determining the most favorable variants of impellers, which were then verified using the numerical modeling methods in the Flow-3D program. 

### 2.3. Numerical Simulations with Flow-3D Program

Testing different rotor impellers using a physical model allows for observing the phenomena taking place while refining. This is a very important step when testing new design solutions without using expensive industrial trials. Another solution is modeling by means of commercial simulation programs such as ANSYS Fluent or Flow-3D [18,19]. Unlike studies on a physical model, in a computer program, the parameters of the refining process and the object itself, including the impeller design, can be easily modified. The simulations were performed with the Flow-3D program version 12.03.02. A three-dimensional system with the same dimensions as in the physical modeling was used in the calculations. The isothermal flow of liquid–gas bubbles was analyzed. As in the physical model, three speeds were adopted in the numerical tests: 200, 300, and 500 rpm. During the initial phase of the simulations, the velocity field around the rotor generated an appropriate direction of motion for the newly produced bubbles. When the required speed was reached, the generation of randomly distributed bubbles around the rotor was started at a rate of 2000 per second. Table 1 lists the most important simulation parameters.

**Figure 6:**
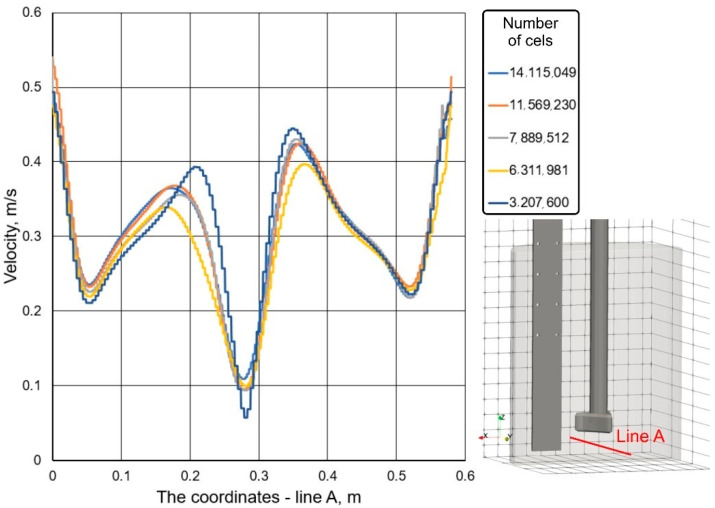
The velocity of the water depending on the size of the computational grid.

In the case of the CFD analysis, the numerical solutions require great care when generating the computational mesh. Therefore, computational mesh tests were performed prior to the CFD calculations. The effect of mesh density was evaluated by taking into account the velocity of water in the tested object on the measurement line A (height of 0.065 m from the bottom) in a characteristic cross-section passing through the object axis (see Figure 6). The mesh contained 3,207,600, 6,311,981, 7,889,512, 11,569,230, and 14,115,049 cells.

The quality of the generated computational meshes was checked using the criterion skewness angle *Q_EAS_* [18]. This criterion is described by the following relationship:

(1) $$Q_{EAS}=\max\left\{\frac{\beta_{\max}-\beta_{eq}}{180-\beta_{eq}},\frac{\beta_{eq}-\beta_{\min}}{\beta_{eq}}\right\},$$

where *β_max_*, *β_min_* are the maximal and minimal angles (in degrees) between the edges of the cell, and *β_eq_* is the angle corresponding to an ideal cell, which for cubic cells is 90°. 

Normalized in the interval [0;1], the value of *Q_EAS_* should not exceed 0.75, which identifies the permissible skewness angle of the generated mesh. For the computed meshes, this value was equal to 0.55–0.65.

Moreover, when generating the computational grids in the studied facility, they were compacted in the areas of the highest gradients of the calculated values, where higher turbulence is to be expected (near the impeller). The obtained results of water velocity in the studied object at constant gas flow rate are shown in Figure 6.

The analysis of the obtained water velocity distributions (see Figure 6) along the line inside the object revealed that, with the density of the grid of nodal points, the velocity changed and its changes for the test cases of 7,889,512, 11,569,230, and 14,115,049 were insignificant. Therefore, it was assumed that a grid containing not less than 7,900,000 (7,889,512) cells would not affect the result of CFD calculations.

**Figure 7:**
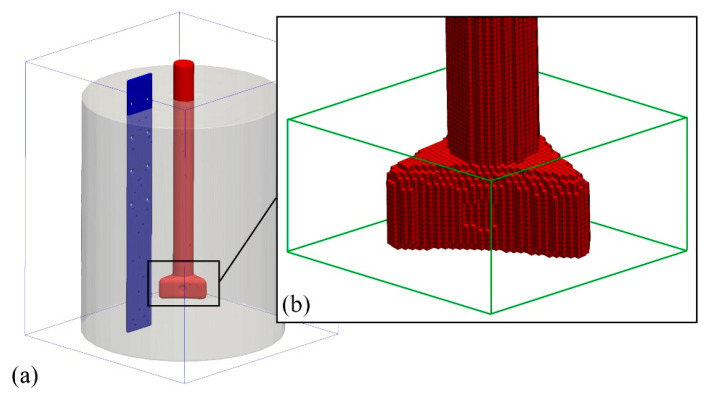
Structured equidistant mesh used in numerical calculations: (**a**) mesh with smoothed, surface cells (the so-called FAVOR method) used in Flow-3D; (**b**) visualization of the applied mesh resolution.

A single-block mesh of regular cells with a size of 0.0034 m was used in the numerical calculations. The total number of cells was approximately 7,900,000 (7,889,512). This grid resolution (see Figure 7) allowed the geometry of the system to be properly represented, maintaining acceptable computation time (about 3 days on a workstation with 2× CPU and 12 computing cores).

The calculations were conducted with an explicit scheme. The timestep was selected by the program automatically and controlled by stability and convergence. From the moment of the initial velocity field generation (start of particle generation), it was 0.0001 s.

When modeling the degassing process, three fluids are present in the system: water, gas supplied through the rotor head (impeller), and the surrounding air. Modeling such a multiphase flow is a numerically very complex issue. The necessity to overcome the liquid backpressure by the gas flowing out from the impeller leads to the formation of numerical instabilities in the volume of fluid (VOF)-based approach used by Flow-3D software. Therefore, a mixed description of the analyzed flow was used here. In this case, water was treated as a continuous medium, while, in the case of gas bubbles, the discrete phase model (DPM) model was applied. The way in which the air surrounding the system was taken into account is later described in detail.

The following additional assumptions were made in the modeling:

— The liquid phase was considered as an incompressible Newtonian fluid.
— The effect of chemical reactions during the refining process was neglected.
— The composition of each phase (gas and liquid) was considered homogeneous; therefore, the viscosity and surface tension were set as constants.
— Only full turbulence existed in the liquid, and the effect of molecular viscosity was neglected.
— The gas bubbles were shaped as perfect spheres.
— The mutual interaction between gas bubbles (particles) was neglected.

#### 2.3.1. Modeling of Liquid Flow

The motion of the real fluid (continuous medium) is described by the Navier–Stokes Equation [20].

(2) $$\frac{du}{\mathrm{dt}}=-\frac{1}{\rho }\nabla p+\nu \nabla^{2}u+\frac{1}{3}\nu \nabla \left(\nabla \cdot \;u\right)+F,$$

where *du/dt* is the time derivative, *u* is the velocity vector, *t* is the time, and *F* is the term accounting for external forces including gravity (unit components denoted by *X*, *Y*, *Z*).

In the simulations, the fluid flow was assumed to be incompressible, in which case the following equation is applicable:

(3) $$\frac{\partial u}{\partial t}+\left(u\cdot \nabla \right)u=-\frac{1}{\rho }\nabla p+\nu \nabla^{2}u+F.$$

Due to the large range of liquid velocities during flows, the turbulence formation process was included in the modeling. For this purpose, the k–ε model turbulence kinetic energy *k* and turbulence dissipation ε were the target parameters, as expressed by the following equations [21]:

(4) $$\frac{\partial \left(\rho k\right)}{\partial t}+\frac{\partial \left(\rho kv_{i}\right)}{\partial x_{i}}=\frac{\partial }{\partial x_{j}}\left[\left(\mu +\frac{\mu_{t}}{\sigma_{k}}\right)\cdot \frac{\partial k}{\partial x_{i}}\right]+G_{k}+G_{b}-\rho \varepsilon -Y_{m}+S_{k},$$

(5) $$\frac{\partial \left(\rho \varepsilon \right)}{\partial t}+\frac{\partial \left(\rho \varepsilon u_{i}\right)}{\partial x_{i}}=\frac{\partial }{\partial x_{j}}\left[\left(\mu +\frac{\mu_{t}}{\sigma_{\varepsilon }}\right)\cdot \frac{\partial k}{\partial x_{i}}\right]+C_{1\varepsilon }\frac{\varepsilon }{k}\left(G_{k}+G_{3\varepsilon }G_{b}\right)+C_{2\varepsilon }\rho \frac{\varepsilon^{2}}{k}+S_{\varepsilon },$$

where *ρ* is the gas density, *σ_κ_* and σ_ε_ are the Prandtl turbulence numbers, *k* and *ε* are constants of 1.0 and 1.3, and *G_k_* and *G_b_* are the kinetic energy of turbulence generated by the average velocity and buoyancy, respectively.

As mentioned earlier, there are two gas phases in the considered problem. In addition to the gas bubbles, which are treated here as particles, there is also air, which surrounds the system. The boundary of phase separation is in this case the free surface of the water. The shape of the free surface can change as a result of the forming velocity field in the liquid. Therefore, it is necessary to use an appropriate approach to free surface tracking. The most commonly used concept in liquid–gas flow modeling is the volume of fluid (VOF) method [22,23], and Flow-3D uses a modified version of this method called TrueVOF. It introduces the concept of the volume fraction of the liquid phase *f_l_*. This parameter can be used for classifying the cells of a discrete grid into areas filled with liquid phase (*f_l_* = 1), gaseous phase, or empty cells (*f_l_* = 0) and those through which the phase separation boundary (*f_l_* ∈ (0, 1)) passes (free surface). To determine the local variations of the liquid phase fraction, it is necessary to solve the following continuity equation:

(6) $$\frac{{\mathrm{df}}_{l}}{\mathrm{dt}}=0.$$

Then, the fluid parameters in the region of coexistence of the two phases (the so-called interface) depend on the volume fraction of each phase.

(7) $$\rho =f_{l}\rho_{l}+\left(1-f_{l}\right)\rho_{g},$$

(8) $$\nu =f_{l}\nu_{l}+\left(1-f_{l}\right)\nu_{g},$$

where indices *l* and *g* refer to the liquid and gaseous phases, respectively.

The parameter of fluid velocity in cells containing both phases is also determined in the same way.

(9) $$u=f_{l}u_{l}+\left(1-f_{l}\right)u_{g}.$$

Since the processes taking place in the surrounding air can be omitted, to speed up the calculations, a single-phase, free-surface model was used. This means that no calculations were performed in the gas cells (they were treated as empty cells). The liquid could fill them freely, and the air surrounding the system was considered by the atmospheric pressure exerted on the free surface. This approach is often used in modeling foundry and metallurgical processes [24].

#### 2.3.2. Modeling of Gas Bubble Flow

As stated, a particle model was used to model bubble flow. Spherical particles (gas bubbles) of a given size were randomly generated in the area marked with green in Figure 7b. In the simulations, the gas bubbles were assumed to have diameters of 0.016 and 0.02 m corresponding to the gas flow rates of 10 and 30 dm^3^·min^−1^, respectively. 

Experimental studies have shown that, as a result of turbulent fluid motion, some of the bubbles may burst, leading to the formation of smaller bubbles, although merging of bubbles into larger groupings may also occur. Therefore, to be able to observe the behavior of bubbles of different sizes (diameter), the calculations generated two additional particle types with diameters twice smaller and twice larger, respectively. The proportion of each species in the system was set to 33.33% (Table 2).

The velocity of the particle results from the generated velocity field (calculated from Equation (3) in the liquid $u_{l}$ around it and its velocity resulting from the buoyancy force $u_{b}$. The effect of particle radius r on the terminal velocity associated with buoyancy force can be determined according to Stokes’ law.

(10) $$u_{b}=\frac{2}{9}\;\frac{\left(\rho_{g}-\rho_{l}\right)}{\mu_{l}}gr^{2},$$

where *g* is the acceleration (9.81).

The DPM model was used for modeling the two-phase (water–air) flow. In this model, the fluid (water) is treated as a continuous phase and described by the Navier–Stokes equation, while gas bubbles are particles flowing in the model fluid (discrete phase). The trajectories of each bubble in the DPM system are calculated at each timestep taking into account the mass forces acting on it. Table 3 characterizes the DPM model used in our own research [18].

## 3. Results and Discussion

### 3.1. Calculations of Power and Mixing Time by the Flowing Gas Bubbles

One of the most important parameters of refining with a rotor is the mixing power induced by the spinning rotor and the outflowing gas bubbles (via impeller). The mixing power of liquid metal in a ladle of height (*h*) by gas injection can be determined from the following relation [15]:

(11) $$\frac{p_{g}}{V_{m}}=\rho \cdot g\cdot u_{B},$$

where *p_g_* is the mixing power, *V_m_* is the volume of liquid metal in the reactor, ρ is the density of liquid aluminum, and *u_B_* is the average speed of bubbles, given below.

(12) $$u_{B}=\frac{n\cdot R\cdot T}{A_{c}\cdot P_{m}\cdot t},$$

where *n* is the number of gas moles, *R* is the gas constant (8.314), *A_c_* is the cross-sectional area of the reactor vessel, *T* is the temperature of liquid aluminum in the reactor, and *P_m_* is the pressure at the middle tank level. The pressure at the middle level of the tank is calculated by a function of the mean logarithmic difference.

(13) $$P_{m}=\frac{\left(P_{a}+\rho \cdot g\cdot h\right)-P_{a}}{\ln\frac{\left(P_{a}+\rho \cdot g\cdot h\right)}{P_{a}}},$$

where *P_a_* is the atmospheric pressure, and *h* is the the height of metal in the reactor. 

Themelis and Goyal [25] developed a model for calculating mixing power delivered by gas injection.

(14) $$p_{g}=2Q\cdot R\cdot T\cdot \ln\left(1+\frac{m\cdot \rho \cdot g\cdot h}{P}\right),$$

where *Q* is the gas flow, and *m* is the mass of liquid metal.

Zhang [26] proposed a model taking into account the temperature difference between gas and alloy (metal).

(15) $$p_{g}=\frac{{\mathrm{QRT}}_{g}}{V_{m}}\left[\ln\left(1+\frac{\rho \cdot g\cdot h}{P_{a}}\right)+\left(1-\frac{T}{T_{g}}\right)\right],$$

where *T_g_* is the gas temperature at the entry point.

Data for calculating the mixing power resulting from inert gas injection into liquid aluminum are given below in Table 4. The design parameters were adopted for the model, the parameters of which are shown in Figure 5.

Table 5 presents the results of mixing power calculations according to the models of Themelis and Goyal and of Zhang for inert gas flows of 10, 20, and 30 dm^3^·min^−1^. The obtained calculation results significantly differed from each other. The difference was an order of magnitude, which indicates that the model is highly inaccurate without considering the temperature of the injected gas. Moreover, the calculations apply to the case when the mixing was performed only by the flowing gas bubbles, without using a rotor, which is a great simplification of the phenomenon.

The mixing time is defined as the time required to achieve 95% complete mixing of liquid metal in the ladle [27,28,29,30]. Table 6 groups together equations for the mixing time according to the models.

**Figure 8:**
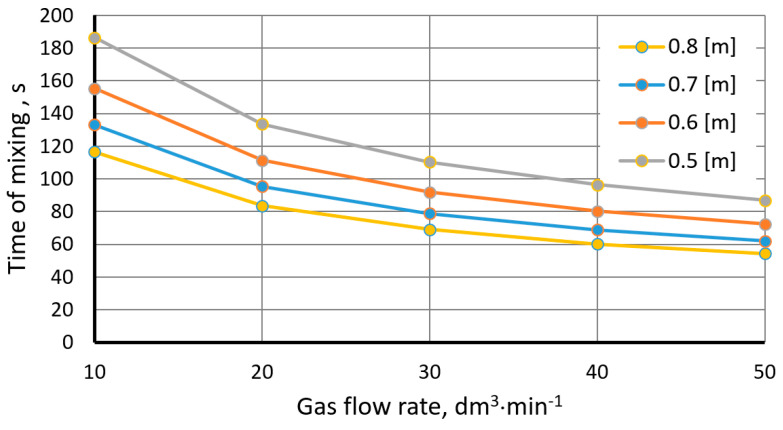
Mixing time as a function of gas flow rate for various heights of the metal column (Iguchi and Nakamura model).

**Figure 9:**
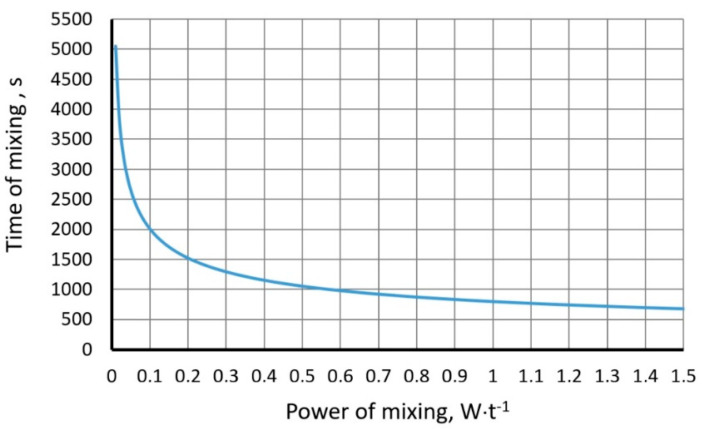
Mixing time as a function of mixing power (Szekly model).

Figure 8 and Figure 9 show the mixing time as a function of gas flow rate for various heights of the liquid column in the ladle and mixing power values.

### 3.2. Determining the Bubble Size

The mechanisms controlling bubble size and mass transfer in an alloy undergoing refining are complex. Strong mixing conditions in the reactor promote impurity mass transfer. In the case of a spinning rotor, the shear force generated by the rotor motion separates the bubbles into smaller bubbles. Rotational speed, mixing force, surface tension, and liquid density have a strong influence on the bubble size. To characterize the kinetic state of the refining process, parameters *k* and *A* were introduced. Parameters *k*, *A*, and *u_B_* can be calculated using the below equations [33].

(16) $$k=2\sqrt{\frac{D\cdot u_{B}}{d_{B}\cdot \pi }},$$

(17) $$A=\frac{6Q\cdot h}{d_{B}\cdot u_{B}},$$

(18) $$u_{B}=1.02\sqrt{g\cdot d_{B},}$$

where *D* is the diffusion coefficient, and d_B_ is the bubble diameter.

After substituting appropriate values, we get

(19) $$d_{B}=3.03\times 10^{4}{\left(\frac{\pi }{D}\right)}^{-2/5}g^{-1/5}h^{4/5}Q^{0.344}N^{-1.48}.$$

**Figure 10:**
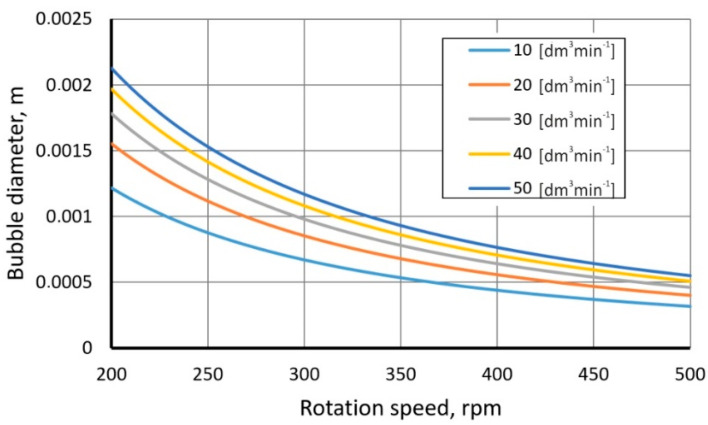
Effect of rotational speed on the bubble diameter.

According to the last equation, the size of the gas bubble decreases with the increasing rotational speed (see Figure 10).

In a flow of given turbulence intensity, the diameter of the bubble does not exceed the maximum size *d*_max_, which is inversely proportional to the rate of kinetic energy dissipation in a viscous flow ε. The size of the gas bubble diameter as a function of the mixing energy, also considering the Weber number and the mixing energy in the negative power, can be determined from the following equations [31,34]:

— Sevik and Park:

(20) $$d_{B\max}={\mathrm{We}}_{\mathrm{kr}}^{0.6}\cdot {\left(\frac{\sigma \cdot 10^{3}}{\rho \cdot 10^{-3}}\right)}^{0.6}\cdot {\left(10\cdot \varepsilon \right)}^{-0.4}\cdot 10^{-2}.$$

— Evans:

(21) $$d_{B\max}={\left[\frac{{\mathrm{We}}_{\mathrm{kr}}\cdot \sigma \cdot 10^{3}}{2\cdot {\left(\rho \cdot 10^{-3}\right)}^{\frac{1}{3}}}\right]}^{\frac{3}{5}}\;\cdot {\left(10\cdot \varepsilon \right)}^{-\frac{2}{5}}\cdot 10^{-2}.$$

The results of calculating the maximum diameter of the bubble *d_Bmax_* determined from Equation (21) are given in Table 7.

### 3.3. Physical Modeling

**Figure 11:**
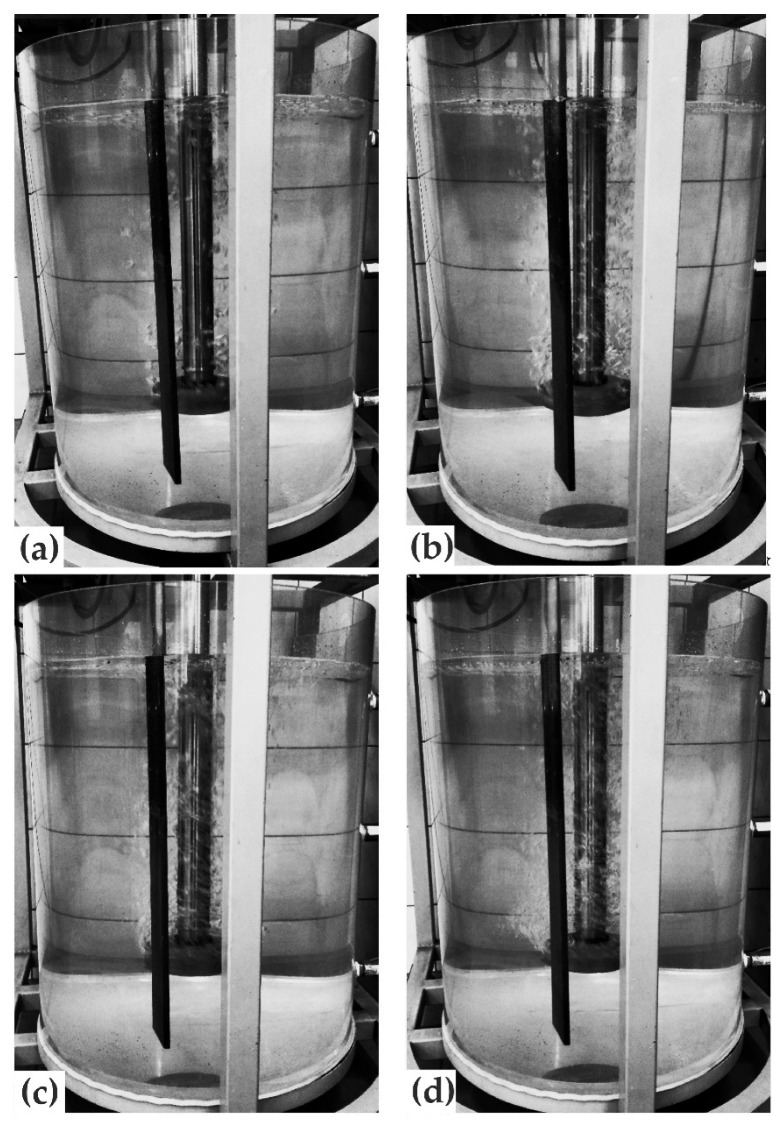
Impeller variant B4—gas bubbles dispersion registered for a gas flow rate of 10 dm^3^·min^−1^ and rotor speed of (**a**) 200, (**b**) 300, (**c**) 400, and (**d**) 500 rpm.

**Figure 12:**
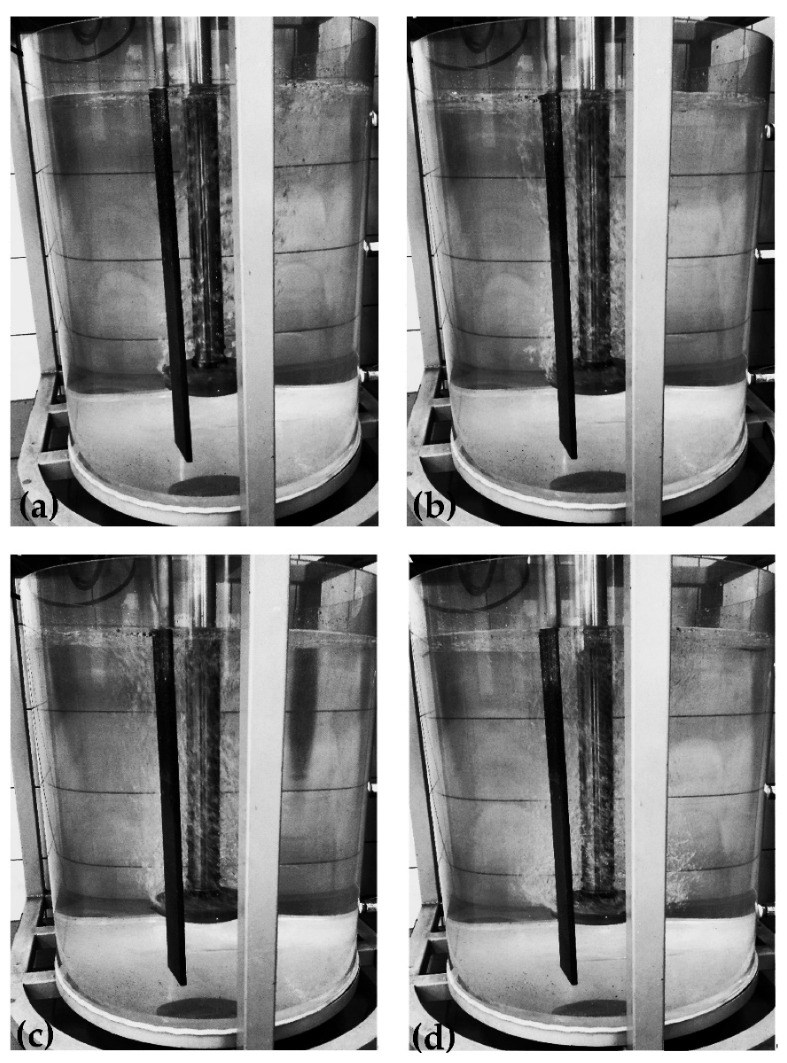
Impeller variant B8—gas bubbles dispersion registered for a gas flow rate of 10 dm^3^·min^−1^ and rotor speed of (**a**) 200, (**b**) 300, (**c**) 400, and (**d**) 500 rpm.

**Figure 13:**
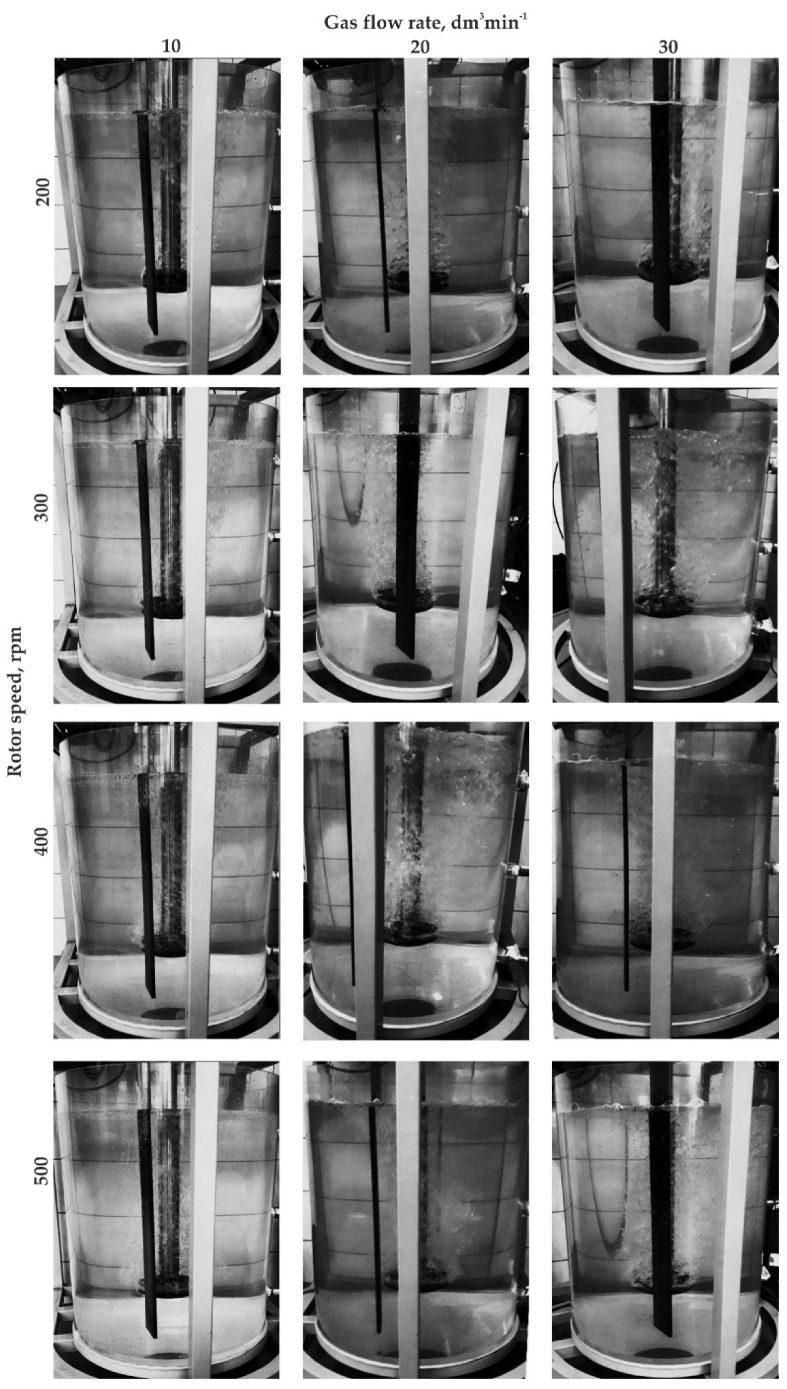
Gas bubble dispersion registered for different processing parameters (impeller variant B12).

**Figure 14:**
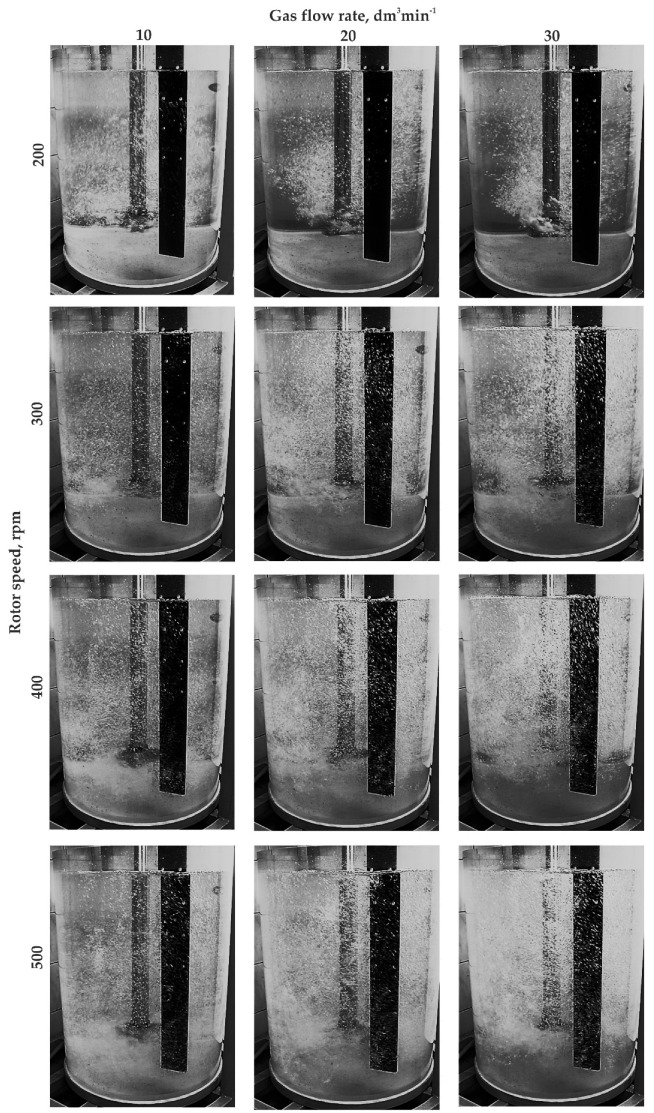
Gas bubble dispersion registered for different processing parameters (impeller variant RT3).

The first stage of experiments (using the URO-200 water model) included conducting experiments with impellers equipped with four, eight, and 12 gas outlets (variants B4, B8, B12). The tests were carried out for different process parameters. Selected results for these experiments are presented in Figure 11, Figure 12, Figure 13 and Figure 14. 

The analysis of the refining variants presented in Figure 11, Figure 12, Figure 13 and Figure 14 reveals that the proposed impellers design model is not useful for the aluminum refining process. The number of gas outlet orifices, rotational speed, and flow did not affect the refining efficiency. In all the variants shown in the figures, very poor dispersion of gas bubbles was observed in the object. The gas bubble flow had a columnar character, and so-called dead zones, i.e., areas where no inert gas bubbles are present, were visible in the analyzed object. Such dead zones were located in the bottom and side zones of the ladle, while the flow of bubbles occurred near the turning rotor. Another negative phenomenon observed was a significant agitation of the water surface due to excessive (rotational) rotor speed and gas flow (see Figure 13, cases 20; 400, 30; 300, 30; 400, and 30; 500).

Research results for a ‘red triangle’ impeller equipped with three gas supply orifices (variant RT3) are presented in Figure 14.

In this impeller design, a uniform degree of bubble dispersion in the entire volume of the modeling fluid was achieved for most cases presented (see Figure 14). In all tested variants, single bubbles were observed in the area of the water surface in the vessel. For variants 20; 200, 30; 200, and 20; 300 shown in Figure 14, the bubble dispersion results were the worst as the so-called dead zones were identified in the area near the bottom and sidewalls of the vessel, which disqualifies these work parameters for further applications. Interestingly, areas where swirls and gas bubble chains formed were identified only for the inert gas flows of 20 and 30 dm^3^·min^−1^ and 200 rpm in the analyzed model. This means that the presented model had the best performance in terms of dispersion of gas bubbles in the model liquid. Its design with sharp edges also differed from previously analyzed models, which is beneficial for gas bubble dispersion, but may interfere with its suitability in industrial conditions due to possible premature wear.

### 3.4. Qualitative Comparison of Research Results (CFD and Physical Model)

**Figure 15:**
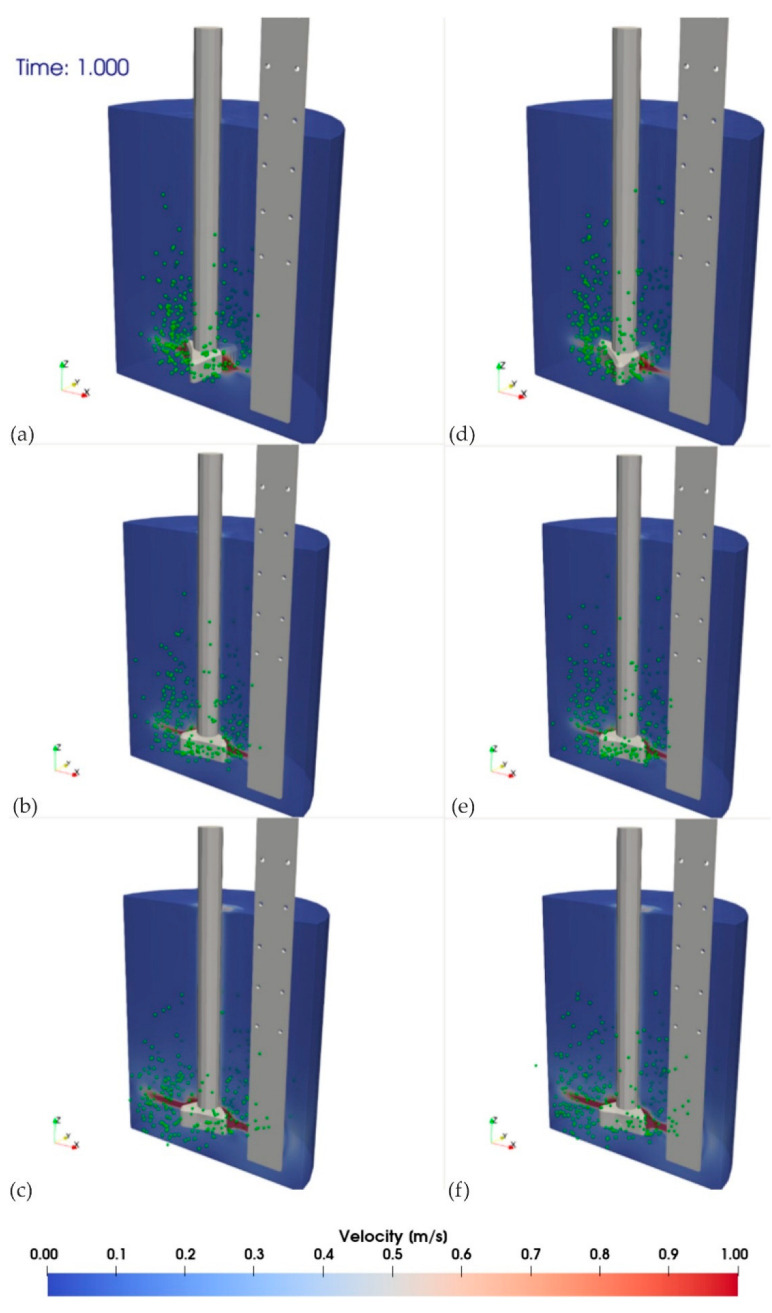
Simulation results of the impeller RT3, for given flows and rotational speeds after a time of 1 s: simulation variants (**a**) A, (**b**) B, (**c**) C, (**d**) D, (**e**) E, and (**f**) F.

**Figure 16:**
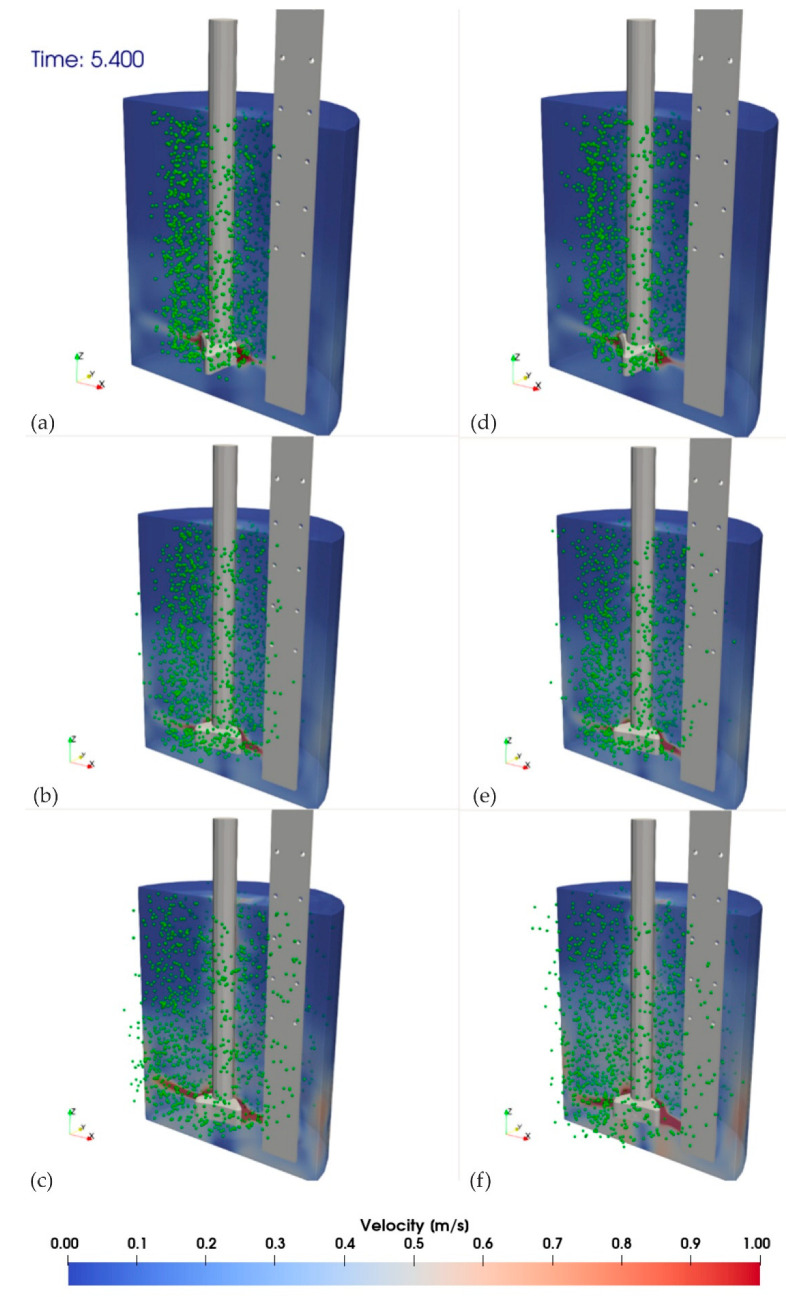
Simulation results of the impeller RT3, for given flows and rotational speeds after a time of 5.4 s.: simulation variants (**a**) A, (**b**) B, (**c**) C, (**d**) D, (**e**) E, and (**f**) F.

The analysis (physical modeling) revealed that the best mixing efficiency results were obtained with the RT3 impeller variant. Therefore, numerical calculations were carried out for the impeller model with three outlet orifices (variant RT3). The CFD results are presented in Figure 15 and Figure 16.

CFD results are presented for all analyzed variants (impeller RT3) at two selected calculation timesteps of 1 and 5.40 s. They show the velocity field of the medium (water) and the dispersion of gas bubbles.

Figure 15 shows the initial refining phase after 1 s of the process. In this case, the gas bubble formation and flow were observed in an area close to contact with the rotor. Figure 16 shows the phase when the dispersion and flow of gas bubbles were advanced in the reactor area of the URO-200 model.

The quantitative evaluation of the obtained results of physical and numerical model tests was based on the comparison of the degree of gas dispersion in the model liquid. The degree of gas bubble dispersion in the volume of the model liquid and the areas of strong turbulent zones formation were evaluated during the analysis of the results of visualization and numerical simulations. These two effects sufficiently characterize the required course of the process from the physical point of view. The known scheme of the below description was adopted as a basic criterion for the evaluation of the degree of dispersion of gas bubbles in the model liquid.

- Minimal dispersion—single bubbles ascending in the region of their formation along the ladle axis; lack of mixing in the whole bath volume.
- Accurate dispersion—single and well-mixed bubbles ascending toward the bath mirror in the region of the ladle axis; no dispersion near the walls and in the lower part of the ladle.
- Uniform dispersion—most desirable; very good mixing of fine bubbles with model liquid.
- Excessive dispersion—bubbles join together to form chains; large turbulence zones; uneven flow of gas.

The numerical simulation results give a good agreement with the experiments performed with the physical model. For all studied variants (used process parameters), the single bubbles were observed in the area of water surface in the vessel. For variants presented in Figure 13 (200 rpm, gas flow 20 and dm^3^·min^−1^) and relevant examples in numerical simulation Figure 16, the worst bubble dispersion results were obtained because the dead zones were identified in the area near the bottom and sidewalls of the vessel, which disqualifies these work parameters for further use. The areas where swirls and gas bubble chains formed were identified only for the inert gas flows of 20 and 30 dm^3^·min^−1^ and 200 rpm in the analyzed model (physical model). This means that the presented impeller model had the best performance in terms of dispersion of gas bubbles in the model liquid. The worst bubble dispersion results were obtained because the dead zones were identified in the area near the bottom and side walls of the vessel, which disqualifies these work parameters for further use. 

**Figure 17:**
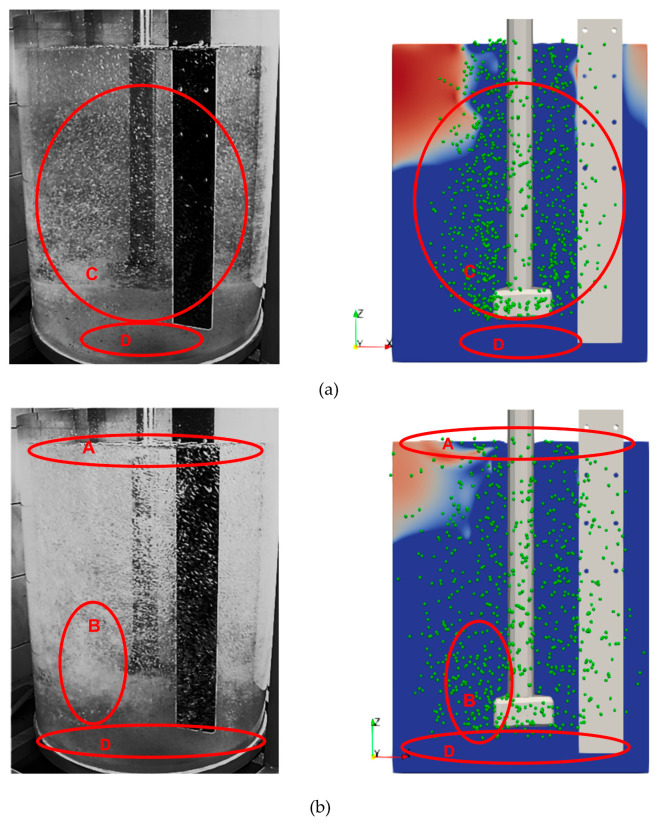
Compilations of model research results (CFD and physical): A—single gas bubbles formed on the surface of the modeling liquid, B—excessive formation of gas chains and swirls, C—uniform distribution of gas bubbles in the entire volume of the tank, and D—dead zones without gas bubbles, no dispersion. (**a**) Variant B; (**b**) variant F.

Figure 17 presents exemplary results of model tests (CFD and physical model) with marked gas bubble dispersion zones. All variants of tests were analogously compared, and this comparison allowed validating the numerical model.

It should be mentioned here that, in numerical simulations, it is necessary to make certain assumptions and simplifications. The calculations assumed three particle size classes (Table 2), which represent the different gas bubbles that form due to different gas flow rates. The maximum number of particles/bubbles (Table 1) generated was assumed in advance and related to the computational capabilities of the computer. Too many particles can also make it difficult to visualize and analyze the results. The size of the particles, of course, affects their behavior during simulation, while, in the figures provided in the article, the bubbles are represented by spheres (visualization of the results) of the same size. Please note that, due to the adopted Lagrangian–Eulerian approach, the simulation did not take into account phenomena such as bubble collapse or fusion. However, the obtained results allow a comprehensive analysis of the behavior of gas bubbles in the system under consideration. 

The comparative analysis of the visualization (quantitative) results obtained with the water model and CFD simulations (see Figure 17) generated a sufficient agreement from the point of view of the trends. A precise quantitative evaluation is difficult to perform because of the lack of a refraction compensating system in the water model. Furthermore, in numerical simulations, it is not possible to determine the geometry of the forming gas bubbles and their interaction with each other as opposed to the visualization in the water model. The use of both research methods is complementary. Thus, a direct comparison of images obtained by the two methods requires appropriate interpretation. However, such an assessment gives the possibility to qualitatively determine the types of the present gas bubble dispersion, thus ultimately validating the CFD results with the water model.

A summary of the visualization results for impellers RT3, i.e., analysis of the occurring gas bubble dispersion types, is presented in Table 8.

Tests carried out for impeller RT3 confirmed the high efficiency of gas bubble distribution in the volume of the tested object at a low inert gas flow rate of 10 dm^3^·min^−1^. The most optimal variant was variant B (300 rpm, 10 dm^3^·min^−1^). However, the other variants A and C (gas flow rate 10 dm^3^·min^−1^) seemed to be favorable for this type of impeller and are recommended for further testing. The above process parameters will be analyzed in detail in a quantitative analysis to be performed on the basis of the obtained efficiency curves of the degassing process (oxygen removal). This analysis will give an unambiguous answer as to which process parameters are the most optimal for this type of impeller; the results are planned for publication in the next article.

It should also be noted here that the high agreement between the results of numerical calculations and physical modelling prompts a conclusion that the proposed approach to the simulation of a degassing process which consists of a single-phase flow model with a free surface and a particle flow model is appropriate. The simulation results enable us to understand how the velocity field in the fluid is formed and to analyze the distribution of gas bubbles in the system. The simulations in Flow-3D software can, therefore, be useful for both the design of the impeller geometry and the selection of process parameters.

## 4. Conclusions

The results of experiments carried out on the physical model of the device for the simulation of barbotage refining of aluminum revealed that the worst results in terms of distribution and dispersion of gas bubbles in the studied object were obtained for the black impellers variants B4, B8, and B12 (multi-orifice impellers—four, eight, and 12 outlet holes, respectively).

In this case, the control of flow, speed, and number of gas exit orifices did not improve the process efficiency, and the developed design did not meet the criteria for industrial tests. In the case of the ‘red triangle’ impeller (variant RT3), uniform gas bubble dispersion was achieved throughout the volume of the modeling fluid for most of the tested variants. The worst bubble dispersion results due to the occurrence of the so-called dead zones in the area near the bottom and sidewalls of the vessel were obtained for the flow variants of 20 dm^3^·min^−1^ and 200 rpm and 30 dm^3^·min^−1^ and 200 rpm. For the analyzed model, areas where swirls and gas bubble chains were formed were found only for the inert gas flow of 20 and 30 dm^3^·min^−1^ and 200 rpm. The model impeller (variant RT3) had the best performance compared to the previously presented impellers in terms of dispersion of gas bubbles in the model liquid. Moreover, its design differed from previously presented models because of its sharp edges. This can be advantageous for gas bubble dispersion, but may negatively affect its suitability in industrial conditions due to premature wearing. 

The CFD simulation results confirmed the results obtained from the experiments performed on the physical model. The numerical simulation of the operation of the ‘red triangle’ impeller model (using Flow-3D software) gave good agreement with the experiments performed on the physical model. This means that the presented model impeller, as compared to other (analyzed) designs, had the best performance in terms of gas bubble dispersion in the model liquid.

In further work, the developed numerical model is planned to be used for CFD simulations of the gas bubble distribution process taking into account physicochemical parameters of liquid aluminum based on industrial tests. Consequently, the obtained results may be implemented in production practice.

## Figures and Tables

**Table 1:** Values of parameters used in the calculations.

Parameter	Value	Unit
Maximum number of gas particles	1,000,000	-
Rate of particle generation	2000	1·s^−1^
Specific gas constant	287.058	J·kg^−1^·K^−1^
Atmospheric pressure	1.013 × 10^5^	Pa
Water density	1000	kg·m^−3^
Water viscosity	0.001	kg·m^−1^·s^−1^
Boundary condition on the walls	No-slip	-
Size of computational cell	0.0034	m

**Table 2:** Data assumed for calculations.

No	Rotor Speed (Rotational Speed) rpm	Bubbles Diameter m	Corresponding Gas Flow Rate dm^3^·min^−1^	No	Rotor Speed (Rotational Speed) rpm	Bubbles Diameter m	Corresponding Gas Flow Rate dm^3^·min^−1^
A	200	0.016	10	D	200	0.02	30
		0.008				0.01	
		0.032				0.04	
B	300	0.016	10	E	300	0.02	30
		0.008				0.01	
		0.032				0.04	
C	500	0.016	10	F	500	0.02	30
		0.008				0.01	
		0.032				0.04	

**Table 3:** Characteristic of the DPM model.

Method	Equations
Euler–Lagrange	Balance equation: $\frac{du_{g}}{dt}=F_{D}\left(u-u_{g}\right)+\frac{g\left(\varrho_{g-}\varrho \right)}{\varrho_{g}}+F.$ *F_D_ (u − u_p_)* denotes the drag forces per mass unit of a bubble, and the expression for the drag coefficient F_D_ is of the form $F_{D}=\frac{18\mu C_{D}Re}{\varrho_{g}^{\cdot }d_{g}^{2}24}.$ The relative Reynolds number has the form $Re\equiv \frac{\rho d_{g}\left|u_{g}-u\right|}{\mu }.$ On the other hand, the force resulting from the additional acceleration of the model fluid has the form $F=\frac{1}{2}\frac{d\rho }{dt\rho_{g}}\left(u-u_{g}\right),$ where *u_g_* is the gas bubble velocity, *u* is the liquid velocity, *d_g_* is the bubble diameter, and *C_D_* is the drag coefficient.

**Table 4:** Data for calculating mixing power introduced by an inert gas.

Parameter	Value	Unit
Height of metal column	0.7	m
Density of aluminum	2375	kg·m^−3^
Process duration	20	s
Gas temperature at the injection site	940	K
Cross-sectional area of ladle	0.448	m^2^
Mass of liquid aluminum	546.25	kg
Volume of ladle	0.23	M^3^
Temperature of liquid aluminum	941.15	K

**Table 5:** Mixing power calculated from mathematical models.

Mathematical Model	Mixing Power (W·t^−1^) for a Given Inert Gas Flow (dm^3^·min^−1^)		
	10	20	30
Themelis and Goyal	11.49	23.33	35.03
Zhang	0.82	1.66	2.49

**Table 6:** Models for calculating mixing time.

Authors	Model	Remarks
Szekely [31]	$\tau =800\varepsilon^{-0.4}$	*ε—*W·t^−1^
Chiti and Paglianti [27]	$\tau =C\frac{V}{Q_{l}}$	*V—*volume of reactor, m^3^ *Ql—*flow intensity, m^3^·s^−1^
Iguchi and Nakamura [32]	$\tau =1200\cdot Q^{-0.4}D^{1.97}h^{-1.0}\upsilon^{0.47}$	*υ—*kinematic viscosity, m^2^·s^−1^ *D—*diameter of ladle, m *h—*height of metal column, m *Q—*liquid flow intensity, m^3^·s^−1^

**Table 7:** The results of calculating the maximum diameter of the bubble using Equation (21).

Model	Mixing Energy ĺ (m^2^·s^−3^)	Weber Number (We_kr_)		
		0.59	1.0	1.2
Zhang and Taniguchi *d*_max_	0.1	0.0167	0.0230	0.026
	0.5	0.0088	0.0121	0.013
	1.0	0.0067	0.0091	0.010
	1.5	0.0057	0.0078	0.009
Sevik and Park *d_B_*_max_	0.1	0.265	0.36	0.41
	0.5	0.139	0.19	0.21
	1.0	0.106	0.14	0.16
	1.5	0.090	0.12	0.14
Evans *d_B_*_max_	0.1	0.247	0.340	0.38
	0.5	0.130	0.178	0.20
	1.0	0.098	0.135	0.15
	1.5	0.084	0.115	0.13

**Table 8:** Summary of visualization results (impeller RT3)—different types of gas bubble dispersion.

No Exp.	A	B	C	D	E	F
Gas flow rate, dm^3^·min^−1^	10			30		
Impeller speed, rpm	200	300	500	200	300	500
Type of dispersion	Accurate	Uniform	Uniform/excessive	Minimal	Excessive	Excessive

## Data Availability

Data are contained within the article.
